# Bioreactor systems for micropropagation of plants: present scenario and future prospects

**DOI:** 10.3389/fpls.2023.1159588

**Published:** 2023-04-19

**Authors:** Hosakatte Niranjana Murthy, Kadanthottu Sebastian Joseph, Kee Yoeup Paek, So Young Park

**Affiliations:** ^1^ Department of Botany, Karnatak University, Dharwad, India; ^2^ Department of Horticultural Science, Chungbuk National University, Cheongju, Republic of Korea; ^3^ Department of Life Sciences, CHRIST (Deemed to be University), Bengaluru, India

**Keywords:** Bioreactor, embryogenesis, immersion culture, *in vitro* propagation, liquid culture, organogenesis, somatic embryo, temporary immersion culture

## Abstract

Plant micropropagation has been adapted in the fields of agriculture, horticulture, forestry, and other related fields for large-scale production of elite plants. The use of liquid media and adoption of bioreactors have escalated the production of healthy plants. Several liquid-phase, gas-phase, temporary immersion, and other modified bioreactors have been used for plant propagation. The design, principle, operational mode, merits, and demerits of various bioreactors used for the regeneration of propagules, such as bulblets, cormlets, rhizomes, microtubers, shoots (subsequent rooting), and somatic embryos, are discussed here. In addition, various parameters that affect plant regeneration are discussed with suitable examples.

## 1 Introduction

Micropropagation of plants is a useful technique for large-scale propagation of crops, medicinal and ornamental plants, tree species, and other economically important plants (Rout et al., 2000; Rout et al., 2006). Micropropagation is a valuable method for rapid production of disease-free plants, rapid multiplication of rare species, genetic transformation of plants, and production of plant-derived bioactive compounds (Rout et al., 2000; Espinosa-Leal et al., 2018). Micropropagation technique involves aseptic culturing of explants, such as apical or axillary meristems, or other parts of the plant body on chemically defined nutrient medium, and maintaining the cultures in controlled environmental conditions for temperature, light, and humidity. The cultured cells from explants are involved in expressing totipotency and morphogenetic responses and follow a definite developmental pathway of dedifferentiation, redifferentiation, and organization of meristematic centers under the influence of phytohormones/growth regulators. Upon expression of totipotency, actively dividing cells induce organogenesis or somatic embryogenesis (Xu and Huang, 2014). Developmental events for the formation of organs or somatic embryos may occur *via* direct and indirect morphogenesis (Rout et al., 2006). Development of shoots from preexisting meristem i.e., regeneration of shoots from apical meristem or axillary meristem is designated as direct organogenesis. Whereas, the regeneration of somatic embryos from single cells or group of cells without the mediation of callus regeneration is referred to as direct embryogenesis. However, in some plants, regeneration of shoots or embryos may develop from the callus regenerated from the cultured explants. Micropropagation involves four concrete stages during organogenesis: initiation of culture, shoot multiplication, induction of roots from regenerated shoots, and acclimatization (Rout et al., 2006). The first stage involves culture initiation when specific and actively growing organs or explants and juveniles are selected and cultured on a well-defined medium. In the second stage, explant cells respond to the stimulus of growth regulators (auxin and cytokinins) and are involved in the division and development of organs (organogenesis). The third stage involves elongation of shoots derived from the multiplication stage, and is subsequently rooted either *ex vitro* or *in vitro*. The fourth stage involves the acclimatization of plants grown *in vitro*, which is a crucial stage in the micropropagation procedure (Rout et al., 2006). Somatic embryogenesis involves five stages: initiation of embryogenesis or induction, during which embryogenic cultures are initiated by culturing the primary explant on a medium supplemented with plant growth regulators, mainly auxin and cytokinin. The second stage involves proliferation of embryogenic cultures on solidified or liquid media supplemented with plant growth regulators, similar to that during initiation. The third stage is pre-maturation of somatic embryos or developmental stage in a medium lacking a plant growth regulator or cytokinin, which stimulates somatic embryo formation and early development. The fourth stage involves the maturation of somatic embryos by culturing on a medium supplemented with abscisic acid and/or reduced osmotic potential (Lema-Ruminska et al., 2013). The fifth stage involves conversion of embryos into plantlets, normally on a medium lacking a plant growth regulator (Arnold et al., 2002). In orchids, *in vitro* cultured explants develop protocorm-like bodies (PLBs) that are regenerated from shoot tips, flower stalk buds, root tips, and leaf segments directly or *via* callus mediation, and are similar to somatic embryos based on their nature and mode of development (Lee et al., 2013). In geophytes, vegetative propagules/organogenic propagules/storage organs, such as bulbs/bulblets (onion, garlic), tubers/microtubers (potato), corms/cormlets (gladiolus, crocus), and rhizomes (ginger, turmeric, lily), are regenerated when different explants are cultured on nutrient medium (Paek and Murthy, 2002; Lian et al., 2003a; Lian et al., 2003b; Jo et al., 2008).

Conventional micropropagation is considered a labor-intensive and costly technology because it involves the maintenance of a large number of culture vessels, semi-solid media, and periodic transfer of plant material to fresh media after subculturing for 4–6 weeks owing to the exhaustion of nutrients in the medium (Maene and Debergh, 1985). Gelling agents substantially raise the expense of *in vitro* production and restrict the extent to which commercial propagation may be automated (Garcia-Ramirez, 2023). Scaled-up and automated methods are consequently preferable to reduce handling throughout the processes necessary for micropropagation, boost multiplication rates, and overcome or minimize production costs (Watt, 2012; Garcia-Ramirez, 2023). Therefore, the use of liquid medium and culture of propagules in bioreactors have evolved as attractive alternatives to conventional propagation methods. The advantages of using liquid medium and bioreactor cultures for micropropagation include: i) a large number of plantlets are easily produced and scaled up; ii) handling of cultures, such as inoculation or harvest, is easy, saving labor and time; iii) cultures are always in contact with the medium, which helps in easy uptake of nutrients and results in stimulation of growth rate; and iv) forced air supply in bioreactor cultures facilitates growth and metabolism of cultured cells and organs (Takayama and Akita, 1994; Paek et al., 2001; Etienne and Berthouly, 2002; Paek et al., 2005). However, liquid cultures have disadvantages, including asphyxia, hyperhydricity, and physiological disorders exhibited by the continuously immersed cultures (Gao et al., 2018; Schuchovski et al., 2020). Several bioreactors designed for microbial cultures cannot be used for plant micropropagation because plant cells and organs may experience shear stress, mechanical damage in stirred tank bioreactors, and foam formation in bubble-aerated bioreactors (Teisson and Alvard, 1999). Therefore, several modifications have been carried out with available bioreactors, and new designs have been developed and adopted exclusively for culturing plant cells and organs, specifically for micropropagation. Temporary immersion, wave, and balloon-type bubble bioreactors have been developed. The design and modification of existing bioreactors for plant micropropagation have been extensively reviewed (Paek et al., 2001; Etienne and Berthouly, 2002; Paek et al., 2005; Watt, 2012; Steingroewer et al., 2013; Mamun et al., 2015; Vidal and Sanchez, 2019). However, bioreactors for regenerating shoots (axillary and adventitious shoots), somatic embryos, and bulbs/corms/micro-tubers/rhizomes require special designs. Different plant species and propagation materials have various requirements that demand specific settings for internal bioreactor environments and determine the most appropriate bioreactor design. The growth and development of plant cells *in vitro* mostly depends on liquid medium circulation, mixing, and aeration for the distribution of oxygen and nutrients (Curtis, 2005; Mamun et al., 2015). The present review focuses on various configurations of bioreactors that have been specifically designed or modified for plant propagation, their advantages, and limitations. Because of the differences in their requirements, the parameters that should be managed in bioreactors for regenerating organogenic propagules, such as bulbs, corms, micro-tubers, rhizomes, shoots, and somatic embryos, have been covered in separate sections.

## 2 Bioreactor systems for plant propagation

Bioreactors are self-contained and sterile environments, which use liquid nutrient media or liquid/air inflow and outflow systems. They are designed for intensive culture and provide the highest opportunity for monitoring and controlling microenvironmental conditions such as agitation, aeration, temperature, and hydrogen ion concentration (pH) (Leathers et al., 1995). The internal environment of a bioreactor is typically controlled at different levels, depending on its model and the plant material. The parameters, including circulation of medium, mixing, aeration, temperature, pH, and dissolved oxygen, are efficiently controlled in bioreactors. Different plant species and propagation materials have different requirements that dictate specific settings for the internal environment of bioreactors and determine the most appropriate bioreactor model to use (Mamun et al., 2015). Based on the environment in the cultivation chamber, bioreactors are broadly classified into four types: liquid-phase, gas-phase, temporary immersion, and modified bioreactors. Liquid-phase bioreactors are of two types: mechanically agitated bioreactors and pneumatically agitated bioreactors based on the mode of circulation of medium inside the container.

### 2.1 Liquid phase bioreactors

Liquid-phase bioreactors are designed such that cultured organs, propagules, or plants are submerged in the liquid medium within the culture vessel. Mixing of the medium and aeration is performed by mechanical stirring using impellers along with an air supply, or mixing is achieved pneumatically by supplying sterile air. Several liquid-phase bioreactors, such as stirred tanks, bubble columns, and airlift bioreactors, adopted for plant propagation are described below.

#### 2.1.1 Mechanically agitated bioreactors

##### 2.1.1.1 Stirred-tank bioreactors

**Figure 1:**
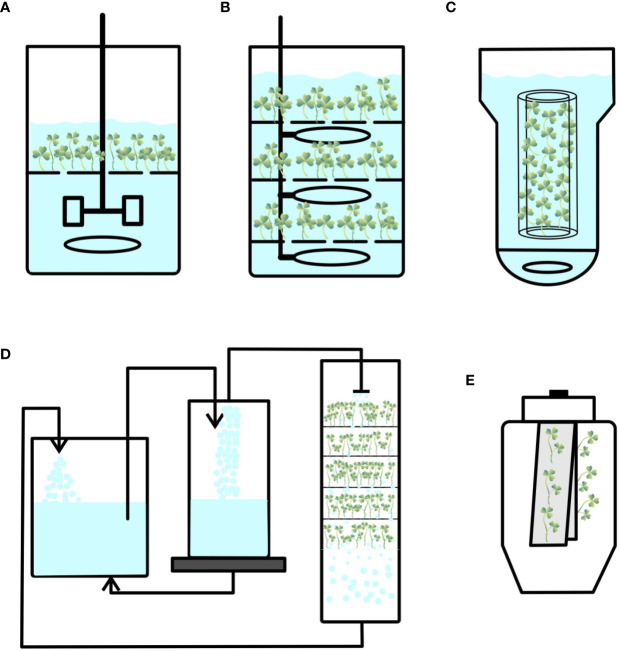
Configurations of different bioreactors used for plant propagation. **(A)** Stirred-tank bioreactor. **(B)** Bubble column bioreactor. **(C)** Airlift bioreactor. **(D)** Nutrient mist bioreactor. **(E)** Trickle bed bioreactor.

Conventional stirred-tank bioreactors consist of an impeller or agitator to stir the medium inside the container (**Figure 1A**). Frequently used stirrers are marine impellers and pinched blade turbines, which create axial fluid flow patterns at low speeds. Various ports for aeration, addition or removal of liquid medium, and distribution of oxygen and nutrients are provided (Mamun et al., 2015). These bioreactors were originally called fermenters and were designed to cultivate microorganisms. They have various advantages in terms of efficient fluid mixing, high oxygen mass transfer, and better control of pH, temperature, dissolved oxygen, and nutrient concentrations, which are ideal for cultivating microorganisms. However, high energy input, shear stress on cultured cells, issues with inoculation and harvesting of biomass, and contamination are the major disadvantages of these bioreactors (**Table 1**). Features such as high specific power input, high energy dissipation rate, turbulence around the agitator, and shear may damage the cultured plant tissues (Huang and McDonald, 2009). Nevertheless, mechanically agitated bioreactors have been used to culture bulblets of *Lilium auratum* and shoot primordia of *Stevia rebaudiana* (Takayama and Akita, 1994; Lim et al., 1998). Severe damage to shoots/propagules have been experienced owing to the high shear stress caused by mechanical agitation. *Daucus carota* somatic embryos have been cultured in stirred-tank reactors (3-L capacity with working volume of 1.7 L) by Jay et al. (1992) to study the effect of dissolved oxygen and pH on embryo growth; however, poor plantlet formation has been observed. Synchronization of growth of developing embryos and a decrease in the viability of harvested embryos have been reported by Ducos et al. (1993). To reduce shear forces, slow-speed stirring bioreactors have been developed by introducing thin silicone tubing hanging inside the periphery of bioreactors (Hvoslef-Eide et al., 2005) and somatic embryos of carrot, Norway spruce, birch, and cyclamen have been cultured. However, none of these modifications have resulted in healthy plantlets.

**Table 1:** Micropropagation of plants *via* organogenesis and somatic embryogenesis: Merits and demerits of bioreactor configurations.

Bioreactor configurations	Merits	Demerits	References
Liquid-phase bioreactors			
Stirred tank bioreactor	-Effective fluid blending. -Substantial oxygen mass transfer. -It is simple to regulate the pH, temperature, dissolved oxygen, and nutrient concentration. -Scaling up is simple. -Alternatives to impellers are offered. -Very flexible in terms of product and manufacturing size.	-As a result of mechanical agitation, high energy costs. -Shear stress on the organs or cells in culture. -Difficulties with biomass harvesting and inoculation -High likelihood of contamination. -Labor-intensive for maintenance, cleaning, and restart.	Georgiev (2014); Mamun et al. (2015); Valdiani et al., (2019)
Bubble column bioreactors	- Simple design since mechanical agitation of the media is not necessary. -Due to the absence of moving parts, maintenance is simpler and the risk of contamination is reduced. -Lower shear stress effects. -Small energy consumption.	-Under high gas flow rates, significant foam formation occurs. -In very viscous fluids, poor fluid mixing occurs. -Separation of gas and liquid in the headspace.	Georgiev (2014); Mamun et al. (2015); Paek et al. (2001); Valdiani et al., (2019)
Airlift bioreactors	-Due to the absence of moving parts, maintenance is simpler and the risk of contamination is reduced. -Better oxygen transport and less shear stress than bubble column bioreactors. -Bubble coalescence is avoided by the medium’s clearly defined flow pattern.	-Under conditions of high gas flow rates, significant foam development occurs. -In excessively viscous fluids, there is poor fluid mixing. -High-density cultures have weak oxygen transport capability.	Georgiev (2014); Mamun et al. (2015); Paek et al. (2001); Valdiani et al., (2019)
Balloon-type bubble bioreactors	-Due to the absence of moving parts, maintenance is simpler and the risk of contamination is reduced. -Superior oxygen transport compared to bubble column bioreactors and less shear stress impact. -Bubble coalescence is avoided by the medium’s clearly defined flow pattern. -Scale-up process is easy	–	Kim et al. (2004); Shohael et al. (2005)
Gas-phase bioreactors			
Nutrient mist bioreactors	-While the liquid phase is supplied as an aerosol containing droplets, the organs are present in the air phase and immobilized on mesh support. -This has the benefit of enhanced gas exchange, increased oxygen and nutrition availability, and decreased shear stress.	-The process of scaling up these bioreactors is challenging.	Georgiev (2014); Mamun et al. (2015); Valdiani et al., (2019)
Trickle-bed bioreactors	- In the air phase, the organs are immobilized on a stainless-steel matrix, and the liquid phase is administered as an aerosol with droplets. -In addition to enhanced gas exchange and decreased shear stress, this has the benefit of increasing oxygen and nutrition availability.	-Scaling up with these bioreactors is challenging.	Georgiev (2014); Mamun et al. (2015); Valdiani et al., (2019)
Temporary immersion bioreactors	-This type of bioreactor enables the cultivation of organs during cycles of immersion or non-immersion. -It operates on the fill-and-drain bioreactor concept, switching between cycles of the liquid and gas phases. -It is not agitated, and cultured organs are not subjected to mechanical stress.	-The scaling-up procedure is challenging with these bioreactors.	Etienne and Berthouly (2002); Georgiev et al. (2014); Mamun et al. (2015); Valdiani et al., (2019); Watt (2012)
Wave-mixed bioreactors	-The disposable bioreactors that use the wave-induced agitation principle are known as “wave bioreactors.” - Aeration parameters were successfully attained. -Contamination and foaming risks are minimal.	-Scaling up these bioreactors is challenging.	Georgiev (2014); Mamun et al. (2015); Valdiani et al., (2019)

#### 2.1.2 Pneumatically agitated bioreactors

##### 2.1.2.1 Bubble column bioreactors

Another conventional bioreactor is the pneumatically agitated bioreactor (**Figure 1B**). It consists of a cylindrical vessel with an air sparger at the bottom of the cylinder, and mixing and agitation are performed by rising air bubbles without any mechanical energy input. These bioreactors are efficient in terms of high heat and mass transfer and involve low operational and maintenance costs (Mamun et al., 2015). The absence of moving parts reduces the risk of contamination (**Table 1**). However, the demerits of these bioreactors are high foam formation under enhanced airflow rates, poor mixing of highly viscous fluids, and gas–liquid separation in the headspace region (Paek et al., 2001).

##### 2.1.2.2 Airlift bioreactors

Another pneumatically agitated bioreactor is the airlift bioreactor that is operated by sparging air into a liquid medium placed at the base of the vessel. It contains an additional draft tube that is used to create an internal or external loop (**Figure 1C**). This creates a high circulation rate and oxygen supply for cultured cells (Mamun et al., 2015). The advantages of these bioreactors are easy maintenance and reduced risk of contamination owing to the absence of mobile parts. This reduces the effect of shear stress and maintains well-defined flow pattern of the medium, and these features are beneficial to produce secondary metabolites from cell suspension cultures (Mamun et al., 2015). The disadvantages of airlift reactors include excessive foam formation under high gas flow rates and relatively poor oxygen transfer capabilities (Paek et al., 2001; Valdiani et al., 2019).

##### 2.1.2.3 Balloon-type bubble bioreactors

**Figure 2:**
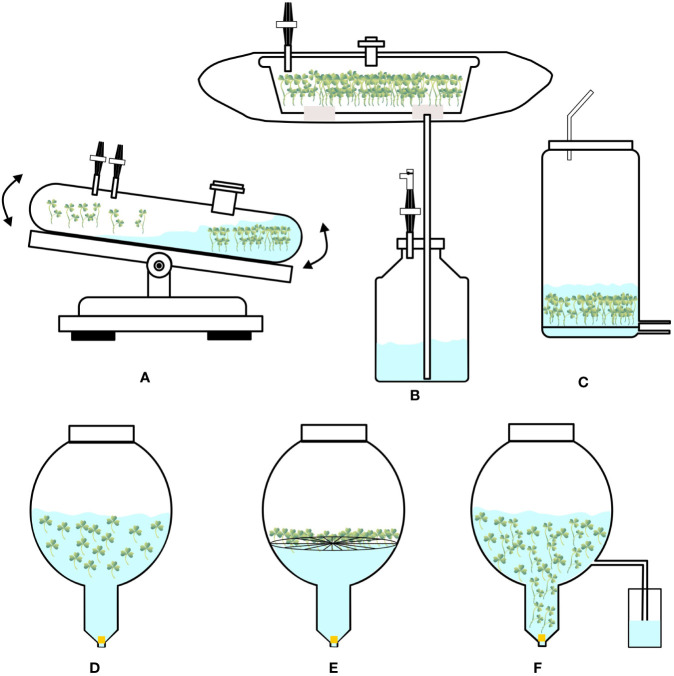
Configurations of **(A)** Wave bioreactor, **(B)** Box-in-bag bioreactor, **(C)** Vertical-column bioreactor, **(D)** Balloon-type bubble bioreactor, **(E)** Balloon-type bubble bioreactor operating on raft method, **(F)** Balloon-type bubble bioreactor operating on ebb and flood method.

These are modified bubble bioreactors (**Figure 2D**) containing balloon-shaped vessels with a concentric tube, with which a circular sparger is installed, and air passing through the sparger and tube helps to lift the cultured biomass at the raiser of the vessel bottom. They have a wide-open area/mouth at the top of a balloon-like cylinder, which helps in easy harvesting of biomass. At the bottom of the vessel, a ‘Y’- or ‘T’- shaped tube helps in collecting sample-suspended cells/biomass and medium. Various probes are used to monitor dissolved oxygen and hydrogen ion concentration (pH). Provision is made with inlet gas to mix oxygen, carbon dioxide, ethylene, or nitrogen with sterile air supplied to the bioreactors. These bioreactors have undergone several modifications for culturing cells, roots, somatic embryos, and organogenic propagules (Paek et al., 2001; Paek et al., 2005). Balloon-type bubble bioreactors are efficient in culturing organogenic propagules/storage organs, such as bulbs/bulblets, pseudobulbs, corms, and rhizomes (**Table 2**). Furthermore, with balloon-type bubble bioreactors, various modes of cultivation have been followed to produce organogenic propagules, such as the complete immersion method and raft method (in the raft method, propagules are grown in the bioreactor with a plastic net or raft; **Figure 2E**), in which the explants are in contact with the liquid medium at the base. In the ebb and flood method, medium is pumped from the storage tank into culture vessels (**Figure 2F**). A series of channels helps in evenly supplying nutrient solution to the plant materials, resulting in uniform growth. The medium remains in the vessel for a few minutes, after which it is drained back to the storage tank for reuse. The drainage process is controlled by a solenoid valve at intervals of 3–6 h depending on the programs set by the timer. In this process, explants/plant materials receive nutrients at regular time intervals at the same time they are in aerated conditions inside culture vessels. Kim et al. (2004) used balloon-type bubble bioreactors (5-L capacity and 4 L liquid medium), followed by immersion, raft, and ebb and flood methods to induce bulblets from shoot tip cultures (to produce virus-free bulblets) of *Allium sativum* (garlic). They could produce 4,049, 5,129, and 3,099 bulblets of varied sizes (0.02–2 gm) after 12 weeks of culture using immersion, raft, and ebb and flood methods, respectively. Balloon-type bubble bioreactors are efficient in culturing somatic embryos and protocorm-like bodies (PLBs; also, somatic embryos of orchids). Shohael et al. (2005) induced somatic embryogenesis in *Eleutherococcus sessiliflorus* and cultured 10 gm embryogenic cells aggregated in 3-L balloon-type bubble bioreactors containing 2-L liquid medium and obtained 128.8 gm mature somatic embryos within four weeks. Subsequently, they cultured 10 gm matured embryos in a liquid medium supplemented with gibberellic acid in balloon-type bubble bioreactors and obtained plantlets after four weeks. Similarly, Yang et al. (2010) induced PLBs in *Oncidium* ‘Sugar Sweet” orchid from shoot tip explants using balloon-type bubble bioreactors. They followed the immersion and ebb and flood methods to cultivate PLBs, and the maximum PLB biomass was obtained in the immersion culture method after 50 days. Furthermore, PLBs regenerated in the bioreactor were successfully developed into the plantlets.

**Table 2:** Selected examples of propagation of bulblets, corms, microtubers, and rhizomes in bioreactor cultures.

Plant species	Regeneration of bulblets/corms/Microtubers/rhizomes	Type of bioreactors used	References
Propagation of bulblets			
*Lilium* oriental hybrids ‘Marcopolo’, ‘Casablanca’	Bulblets	Balloon-type bubble bioreactors	Lim et al. (1998)
*Lilium* oriental hybrid ‘Casablanca’	Bulblets	Balloon-type bubble bioreactors	Lian et al. (2002)
*Lilium* oriental hybrid *‘*Casablanca*’*	Bulblets	Balloon-type bubble bioreactors	Lian et al. (2003a)
*Allium sativum*	Bulblets	Balloon-type bubble bioreactors	Kim et al. (2004)
*Lilium* hybrids	Bulblets	Balloon-type bubble bioreactors	Lian et al. (2014)
Propagation of pseudobulbs			
*Bletilla striata*	Pseudobulbs	Temporary immersion bioreactor system	Zhang et al. (2018)
Propagation of corms			
*Crocus sativus*	Corms	Balloon-type bubble bioreactors	Dewir et al. (2022)
*Alocasia amazonica*	Corms	Balloon-type bubble bioreactors	Jo et al. (2008)
Propagation of microtubers			
*Solanum tuberosum* cv. Yukishiro	Microtubers	Jar fermenters/bioreactors (Immersion and temporary immersion)	Akita and Takayama (1994)
*Solanum tuberosum* ‘Zhongshu-3’	Microtubers	Nutrient mist bioreactors	Hao et al. (1998)
*Solanum tuberosum* ‘Bintje’, ‘Desiree’ and ‘Ostara’	Microtubers	Temporary immersion cultures – RITA bioreactors	Teisson and Alvard (1999)
*Solanum tuberosum* ‘Desiree’ and ‘Atlantic’	Microtubers	Temporary immersion bioreactors	Jimenez et al. (1999)
*Solanum tuberosum* ‘Russet Burnank’	Microtubers	Rotating bottle bioreactors on rollers	Yu et al. (2000)
*Solanum tuberosum* ‘Atlantic’	Microtubers	Vertical column airlift bioreactors – Continuous immersion and Temporary immersion	Piao et al. (2003)
Propagation of rhizomes			
*Cymbidium sinense*	Rhizomes	Balloon-type bubble bioreactors (continuous immersion and temporary immersion)	Gao et al. (2014)

### 2.2 Gas-phase bioreactors

In gas-phase bioreactors, cultured explants are maintained in ambient sterile air conditions and are intermittently supplied with liquid medium in the form of bubbles, spray, or mist. Gas-phase bioreactors were originally developed for hairy root cultures to overcome the limitations of oxygen supply, shear stress, and physical and physiological problems experienced by cultured roots, and such reactors were also used for plant propagation. The merits and demerits of gas-phase bioreactors for cultivation of organogenic propagules and somatic embryos are explained below.

#### 2.2.1 Nutrient mist bioreactors

Nutrient mist bioreactors are gas-phase bioreactors that provide ***in vitro*** plants with small droplets of culture medium fully infused with sterile gas generated as an aerosol into the growth chamber (**Figure 1D**). Mist bioreactors with various configurations promote growth with increased shoot and somatic embryogenesis. Correll and Weathers (2001a; 2001b) propagated *Dianthus* plants that were cultured in mist chambers with well-rooted plantlets and obtained quality plants with good physiological characteristics. Fei and Weathers (2014) cultured embryogenic cells of *Daucus carrota* (carrot) in a mist bioreactor and regenerated plants in a single culture system from early-stage embryos to germinated plantlets. Mist bioreactors offer excellent growth of embryos and plantlets when the liquid medium is delivered as an aerosol with droplets on the immobilized mesh. This offers the advantages of improved gas exchange, oxygen supply, and nutrient availability inside the growth chamber. However, such bioreactors have the disadvantages of scale up (**Table 1**).

#### 2.2.2 Trickle-bed bioreactors

In this type of bioreactors, medium is usually supplied from the top of the culture vessel through nozzles integrated into the headspace (Kim et al., 2002; Kuzma et al., 2009). Medium is supplied in the form of droplets that trickle over growing explants (shoots/somatic embryo biomass) that are maintained on the mesh or support. Spent medium is drained from the bottom of the bioreactor to a reservoir, and medium is recirculated at specific time intervals and rates (**Figure 1E**). Szopa et al. (2019) tested various systems, such as a cone bioreactor (continuous immersion system), cylindrical tube bioreactor (continuous immersion system), nutrient sprinkle bioreactor, and temporary immersion bioreactor (RITA and Plantform), to produce microshoots of *Schisandara chinensis*. Among them, culture in nutrient sprinkle bioreactor yielded the highest accumulated biomass. These results indicate that nutrient sprinkler bioreactors can also be used for shoot propagation. A disadvantage of these bioreactors is the difficulty in scaling up cultures (**Table 1**).

#### 2.2.3 Temporary immersion bioreactors

Temporary immersion bioreactors or temporary immersion systems (TIS) are designed for periodic immersion of cultured plant tissues or propagules in liquid medium, followed by draining and exposing plant tissues to a sterile gaseous environment. Majority of TIS contains two or more compartments in the container or separate vessels; medium is pushed from a reservoir compartment/vessel to a compartment/vessel in which explants or plants are cultured. Usually, immersion period can be programmed from a few minutes to a few hours. An air pump and solenoid valve are used to circulate the medium. An automated TIS was first developed by Tisserat and Vandercook (1985), and was designated as an automated plant culture system, in which shoot tips of orchids, aster, cow tree, callus of date palm, and carrot were cultured. Better growth of plants and calli was noticed in the automated system than in the manual system. Subsequently, several systems were developed by Aitken-Christie and Davies (1988); Simonton et al. (1991); Alvard et al. (1993, RITA system), and Escalona et al. (1999, twin flask or BIT system). TISs are free from contamination and help propagules with an adequate supply of nutrients. They can overcome the issues of hyperhydricity in cultured plant tissues by creating conditions for optimal humidity (Alabarran et al., 2005). The gaseous environment in TISs overcomes the issue of oxygen limitation. Furthermore, excellent growth and involvement of propagules can be achieved by enriching the percentage of CO_2_ and light intensity in culture vessels (Aragon et al., 2005; Aragon et al., 2010; Curtis and Tuerk, 2008). Trauger et al. (2022) also showed how CO_2_ enrichment affected the growth of cocoa somatic embryos and yam nodal cultures. According to their findings, increased CO_2_ during plant propagation considerably enhanced the development of cocoa and yam propagules and eliminated the requirement for added sugars in the tissue culture growth medium. The designs and operations of various TISs are briefly described here, and detailed configurations of TIS, principles, and technological advances have been presented in previous reviews (Etienne and Berthouly, 2002; Afreen, 2006; Watt, 2012; Georgiev et al., 2014; Garcia-Ramirez, 2023).

##### 2.2.3.1 Twin-flask system

**Figure 3:**
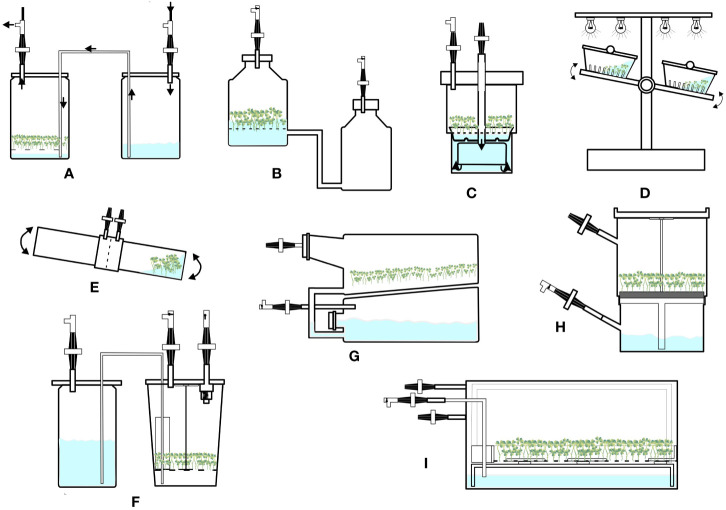
Configurations of temporary immersion bioreactors. **(A)** Twin flask bioreactor. **(B)** Ebb and flow bioreactor. **(C)** RITA bioreactor. **(D)** Rocker system. **(E)** Bio-MINT bioreactor. **(F)** RALM bioreactor. **(G)** SETIS bioreactor. **(H)** PLANTIMA system. **(I)** PLANTFORM bioreactor.

The twin-flask system consists of two vessels connected by silicone tubing (**Figure 3A**). One vessel works as a culture chamber, and the other is a medium reservoir. The vessels are connected to airlines and controlled by timers and solenoid valves. This system maintains constant sterility and allows easy cultivation of plant propagules for a long period. Demerits of this system include a lack of operation for renewal of growth medium, forced ventilation, and automation processes (Georgiev et al., 2014). Shoots, nodule clusters, and embryos have been cultivated using this system. For example, Niemenak et al. (2008) cultured somatic embryos of *Theobroma cacao* in a twin-flask system, and subsequently, matured embryos were converted into plants after sowing. Recently, Orozco-Ortiz et al. (2023) tested three temporary immersion systems namely RITA, SETIS, and BIT systems in addition to a semisolid medium for the multiplication of sugarcane (*Saccharum* spp. variety LAICA 04-809) shoots. Of all the temporary immersion cultures BIT bioreactor generated the highest quality of shoots without hyperhydricity.

##### 2.2.3.2 Ebb and flow system

This system consists of two vessels interconnected by silicone tubing; the larger vessel acts as a culture vessel, and the smaller vessel is used for storing liquid medium or as a medium reservoir (**Figure 3B**). Medium is supplied from the reservoir to the main culture vessel using an air pump, solenoid valve, and timer. With the main vessel, a plastic or polypropylene net is sometimes used as a raft to hold materials away from the container. The main vessel is aerated using sterile air through a sparger during immersion period. This system is simple and reliable; however, supplementation of gases, such as CO_2_ and ethylene, is a limitation of these bioreactors (Georgiev et al., 2014). This system has been used to propagate shoots, bulblets, cormlets, PLBs, and regeneration plants (Chakrabarty et al., 2003; Lian et al., 2003a; b; Jo et al., 2008, Park et al., 2000). Lian et al. (2003a) compared semisolid cultures with liquid cultures using an ebb and flow system for propagation of *Lilium* bulblets and obtained 81 and 1,025 bulblets respectively. These results demonstrate the superiority of the ebb and flow system over semi-solid cultures.

##### 2.2.3.3 RITA

The Recipient for Automated Temporary Immersion system (RITA) was developed by Teisson and Alvard (1995) (supplied by VITROPIC, France), which is a single autoclavable polypropylene vessel with two compartments (**Figure 3C**). The upper compartment is the culture vessel, and the lower one is the medium reservoir. The two compartments are interconnected, and flow of medium is controlled by a solenoid valve and timer. These systems are convenient for handling and culturing; however, no provisions for medium renewal and forced ventilation are some issues (Georgiev et al., 2014). RITA has been used for the propagation of plants such as lingonberry, lowbush blueberry, and plum (Debnath, 2008; Debnath, 2009; Arigundam et al., 2020; Gogo et al., 2022). However, some plants regenerated in this system develop hyperhydricity; therefore, this system requires further improvement of air circulation.

##### 2.2.3.4 Rocker system

In this system, culture vessels or boxes are kept on a platform that tilts by mechanical energy. Explants are normally placed directly into the culture vessel or placed using different support materials, such as nets, rock wool cubes, and polyurethane foam. Medium reaches the explants through mechanical moment of the entire vessel (**Figure 3D**). The cultivation vessels or boxes are made of polycarbonate and are usually rectangular in shape with lateral mouth openings. These systems might be useful in some types of plant cultures; however, they are electromagnetically driven and involve energy and investment costs (Georgiev et al., 2014). A liquid Lab rocker system is used to produce microtubers in potatoes (Kamarainen-Karppinen et al., 2010).

The newly developed BioMINT systems consist of two cylindrical polycarbonate vessels that are fixed opposite to each other (**Figure 3E**). Of the two vessels, one is used for culturing explants/plants, and the other is the medium reservoir. Medium flows through adaptors from one to another vessel and facilitates imbibing of cultures alternately. CO_2_ enrichment is accomplished through a port. These systems have been used for *in vitro* proliferation and elongation of pepper shoots (Bello-Bello et al., 2010) and micropropagation of Cedar plants (Pena-Ramirez et al., 2010).

##### 2.2.3.5 RALM, SETIS, PLANTIMA, and PLANTFORM bioreactors

RALM (Ralm Industries, Brazil) (**Figure 3F**) is a twin-flask system. SETIS (Vervit, Belgium) (**Figure 3G**) operates on the principle of an ebb and flow system. PLANTIMA (A-Tech Bioscientific Co., Ltd., Taiwan) (**Figure 3H**) and PLANTFORM (Plant Form AB, Sweden & TC propagation Ltd., Ireland) (**Figure 3I**) work on the principle followed by RITA. These systems have been used to propagate plants such as *Dioscorea alata* (Yan et al., 2011), *Siraitia grosvenorii* (Yan et al., 2010), *Chrysanthemum morifolium*, *Fragaria* x *ananassa*, and *Cnidium officinale* (Hwang et al., 2022).

#### 2.2.4 Wave bioreactors

The idea behind wave bioreactors is wave-induced agitation of cultures. This device loads culture bags onto a rocking platform that generates waves inside the bags to enable the contents (medium and cultured propagules) to mix and shake and facilitate a liquid or gaseous environment (Eibl et al., 2009; Eibl et al., 2010; **Figure 2A**). Changes can be made to the operating conditions such as the rocking rate, angle, aeration rate, and dimension of culture bags. The term “disposable bioreactors” refers to culture bags that are typically composed of polyethylene, polystyrene, polytetrafluoroethylene, polypropylene, or ethylene vinyl acetate and are delivered in a presterilized state (Eibl et al., 2010). Without foaming and shear stress, aeration and oxygen transfer may be effectively accomplished in these systems. Online monitoring options are available for temperature, pH, and dissolved oxygen. However, these bioreactors are expensive.

The modified version called “Box-in-bag” temporary immersion system (10-L capacity) has been developed by Ducos et al. (2009) specifically to propagate *Coffea canephora* by culturing somatic embryos. The bags are made of a transparent film composed of polyethylene and nylon and are supplied closed on three sides. They are 750 × 420 mm in size and have two polyphenylene ports molded into the film (one above and another below). A rigid transparent box of 50 × 30 × 10 cm made of polycarbonate is kept inside the bag. Port A is connected to the inoculum chamber, and port B is connected to the medium reservoir below (**Figure 2B**). The entire system is kept in a culture room and is loaded with 5 L culture medium along with the inoculum. Ducos et al. (2009) have used these bioreactors to convert torpedo-stage embryos into plantlets after germination.

### 2.3 Modified bioreactors

These are vertical column-shaped bioreactors with a capacity of 10 L, in which a circular sparger is placed at the bottom of the bioreactors, and sterile air is pumped from the basal region of the bioreactors (**Figure 2C**). These are modified bubble column bioreactors, in which a net is provided above the sparger to place propagules/explants. Ports are used for inlet and outlet of medium at the base of culture vessels. Additional ports are available for supplying gases, such as CO_2_, and they can be easily modified to work on the ebb and flow principles. These bioreactors are quite useful in cultivating plants that can grow vertically, and nodal cuttings can be prepared for *ex vitro* rooting of shoots from these *in vitro* grown plants. Such bioreactors are useful in producing chrysanthemum shoots (Hahn and Paek, 2005) and microtubers in potatoes (Piao et al., 2003).

## 3 Factors affecting the growth and differentiation of propagules in bioreactors

Micropropagation of plants *in vitro* is affected by several factors, including selection and source of explants, selection of medium, optimization of chemical factors of medium, such as macroelements and microelements, concentration and type of sugars, type and concentration of phytohormones, and physical factors such as light, photoperiod, temperature, and humidity; these factors should be optimized for particular species considered for regeneration by using semisolid cultures. Bioreactors offer some advantages for culturing propagules, particularly, large volumes for cultivation, providing nutrients optimally to the developing propagules, and facilitating physical conditions that are involved in morphogenetic events. Micropropagation is achieved in bioreactors mostly in batch mode; however, the mode of differentiation and development, i.e., the process involved in multiplication of propagules, shoots, and somatic embryos, varies in different plant species. For example, cultured explants may be involved in the differentiation of bulblets, cormlets, or rhizomes, as in the case of onions, garlic, lilies, and orchids. Similarly, explants may be involved in regenerating axillary or adventitious shoot and the subsequent rooting of shoots, as in the case of several crops and ornamentals and medicinal plants. Some plants propagate *via* somatic embryogenesis that involves the induction, differentiation, maturation, and germination of embryos. Therefore, depending on the mode of regeneration, operating parameters, such as replenishment of medium (control of medium factors), mode of operation (continuous immersion, periodic immersion, or deep flow method), substrate or inoculum density, control of the gaseous environment (oxygen, carbon dioxide, and ethylene), shear stress, and light are essential, even in bioreactor cultures.

### 3.1 Factors affecting the growth of bulblets, cormlets, rhizomes, and microutbers

Various factors affect the regeneration of propagules in bioreactor cultures; therefore, selection of bioreactor and mode of operation are very important. Lian et al. (2014) investigated the regeneration of bulblets from the bulb-scale segments of various *Lilium* cultivars. They selected 5-L balloon-type bubble bioreactors with 1 L Murashige and Skoog medium supplemented with 0.3 mg·L^-1^ naphthalene acetic acid, 1 mg·L^-1^ benzyl adenine, and 30 gm·L^-1^ sucrose and adopted continuous immersion and ebb and flow for regeneration of bulblets of *Lilium* ‘Casa Blanca’. No bulblet regeneration was observed with continuous immersion, whereas in the ebb and flow method, explants developed bulblet regeneration. Furthermore, they tested the effects of the number (four, six, and eight times per day for 30 min immersion in liquid medium) and duration (four times per day for 15, 30, 60, and 120 min) of medium supply on the regeneration of bulblets with ebb and flow type bioreactors. They reported the highest percentage of bulblet formation (75.8%) when medium was supplied four times a day for 15 min.

**Figure 4:**
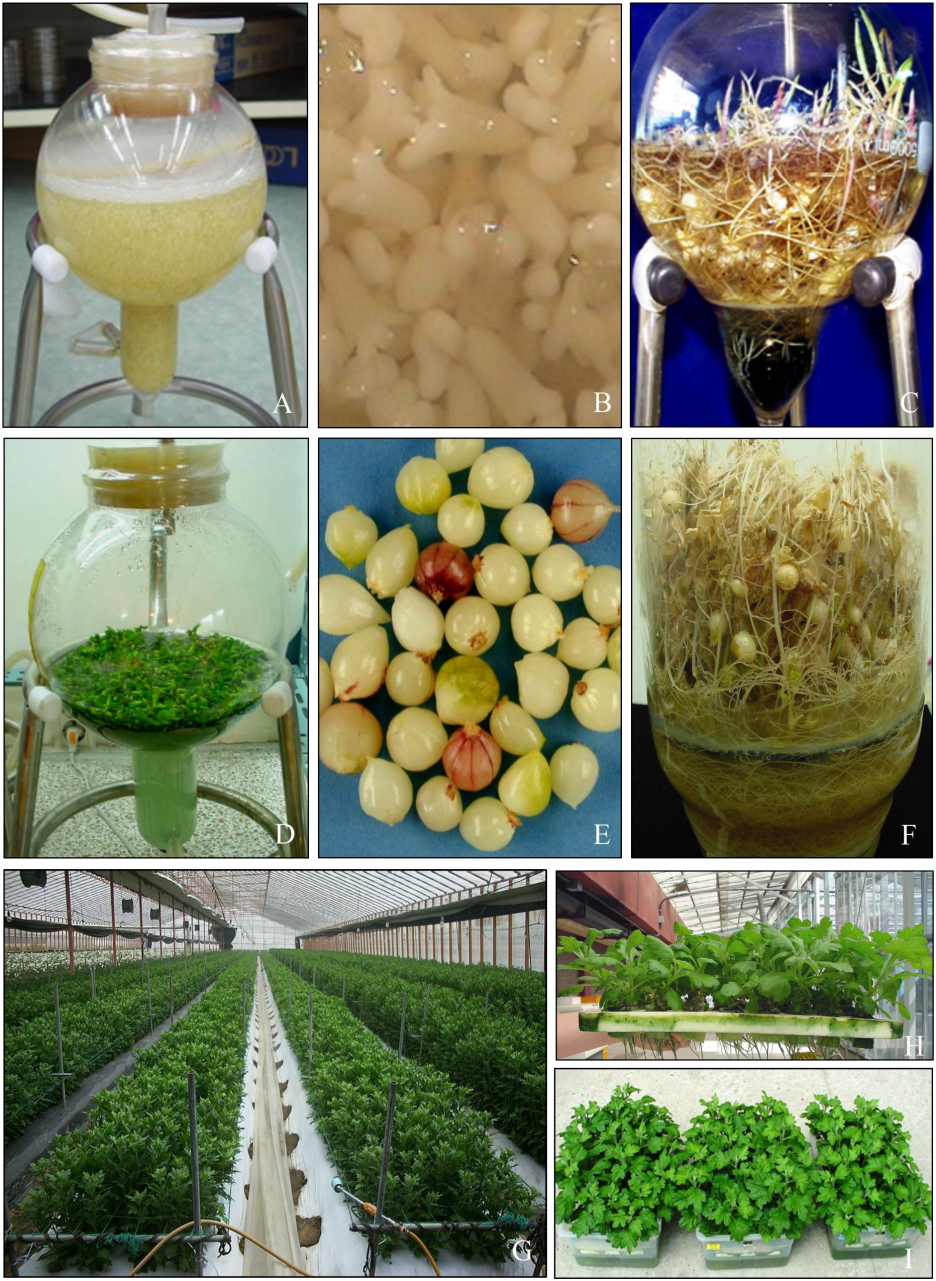
Application of bioreactors for micropropagation. **(A, B)** Somatic embryos of Siberian ginseng cultured in balloon-type bubble bioreactor. **(C)** *Lilium* bulblets cultured in balloon-type bubble bioreactor. **(D)** *Phalaenopsis* PLBs cultured in balloon-type bubble bioreactor with raft system. **(E)** Garlic bulblets harvested from balloon-type bubble bioreactor. **(F)** Potato microtubers developed in vertical column bioreactors. **(G)** Chrysanthemum plants in the greenhouse. **(H, I)** Chrysanthemum plantlets after *ex vitro* rooting by hydroponic method and direct planting to the potting medium by dipping in indole acetic acid powder.

Lian et al. (2014) conducted several experiments on *Lilium* bulblet enlargement/growth after induction *in vitro*, because bulblets that had reached a minimum size of 1 gm could successfully acclimatize after transplantation of potting medium. They cultured 0.1-gm-sized bulblets in Balloon-type bubble bioreactors (BTBBs) using continuous immersion and temporary immersion (ebb and flow) and reported that a continuous immersion system was superior to temporary immersion during the bulblet enlargement/growth stage (**Figure 4C**). Lian et al. (2014) reported that inoculation density, aeration, temperature, and light intensity had a profound influence on growth (enlargement of bulblets) in bioreactor cultures. Enlargement of *Lilium* bulblets *in vitro* in liquid medium/culture is a lengthy process and takes 16 weeks. They also studied the kinetics of nutrient uptake by bulblets during bioreactor cultures and observed depletion of several minerals and sugars in the medium every four weeks. Therefore, they adopted the fed-batch culture method, that is, supplementation of fresh medium with cultures, and could mass-propagate several *Lilium* cultivars.

Piao et al. (2003) tested three different bioreactor systems, including temporary immersion using ebb and flow, continuous immersion with net/raft, or without a net, to produce microtubers of potato ‘Atlantic’. Again, factors, such as inoculum density and medium exchange, affected tuber size during shoot multiplication and tuber induction stages. **Table 2** presents several examples of regeneration of bulblets, corms/cormlets, minitubers, and rhizomes in bioreactor cultures.

### 3.2 Factors affecting the growth of axillary and adventitious shoots

Regeneration of axillary and adventitious shoots has been achieved in various plant species using bioreactor systems (**Table 3**), and such regenerated shoots have been used for *in vitro* or *ex vitro* rooting. However, in certain other plant species, shoot induction and root regeneration has been simultaneously achieved (Jeon et al., 2009; Thul and Kukreja, 2010). Among various bioreactors, temporary immersion systems have been found to be suitable for shoot regeneration, and continuous immersion systems have not been found to be suitable for axillary or adventitious shoot regeneration because these systems induce asphyxia and hyperhydricity of regenerated shoots (Etienne and Berthouly, 2002; Chakrabarty et al., 2003). Varied factors such as inoculum density, aeration volume, and light intensity that affect shoot regeneration should be thoroughly analyzed in certain bioreactor systems. For example, Jin et al. (2013) applied balloon-type bubble bioreactors to propagate grape rootstocks and analyzed the effects of inoculum density, air volume/aeration, and light intensity on plant growth. Inoculum density is an important physical parameter that influences growth during micropropagation. An optimum inoculation density is necessary for dynamic growth and proliferation of cultures *in vitro*. Hahn and Paek (2005) found 80 nodes as the best inoculum density for shoot multiplication of chrysanthemum in bioreactors. Piao et al. (2003) reported the maximal growth of potatoes when 50 nodal explants were cultured in a bioreactor. Similarly, Jin et al. (2013) reported that an initial inoculum density of 65 nodes was suitable for regenerating axillary shoots. Air volume is responsible for medium circulation and oxygen transfer in explants (Wu et al., 2011). A lower aeration volume may be responsible for oxygen deficiency, and higher aeration has been reported to be owing to the stripping off of essential gases such as carbon dioxide and ethylene (Gao and Lee, 1992). An air volume of 150 mL·min^-1^ was found to be appropriate for shoot regeneration in nodal cultures of grape with varied air volumes (50, 100, 150, and 200 mL·min^-1^). Light intensity is another important factor affecting the growth and photosynthesis of cultured propagules (Mukherjee et al., 2010). Of various light intensities (30, 50, and 70 µmol·m^-2^·s^-1^) tested by Jin et al. (2013), 50 µmol·m^-2^·s^-1^ was found to be suitable for regeneration of grape plants using balloon-type bubble bioreactors. RITA and SETIS temporary immersion cultures have been used to regenerate horticultural and medicinal plants (Monja-Mio et al., 2021; Hwang et al., 2022).

**Table 3:** Selected examples of shoot proliferation and multiplication in bioreactor cultures.

Plant species	Shoots proliferation and multiplication	Type of bioreactors used	References
Shoot multiplication			
*Chrysanthemum* (*Dendranthema* *grandiflorum* Kitam ‘Cheonsu’)	Multiplication of shoots	Vertical column bioreactors with ebb and flood system or Deep flow technique	Hahn and Paek (2005)
*Malus domestica* rootstock M.9 EMLA	Multiplication of shoots	Balloon-type bubble bioreactors (continuous immersion and temporary immersion)	Chakrabarty et al. (2003; Kim et al., 2020)
Shoot multiplication and regeneration of plants			
*Vitis flexusa*	Multiplication of shoots and regeneration of plants	Balloon-type bubble bioreactors (with net/raft)	Park et al. (2015)
*Vitis vanifera* rootstock 5BB	Multiplication of shoots and regeneration of plants	Balloon-type bubble bioreactors (with net/raft)	Jin et al. (2013)
*Castanea sativa* x *C. crenata*	Multiplication of shoots and regeneration of plants	RITA and plantform temporary immersion cultures	Vidal et al. (2015)
*Castanea sativa x C. crenata*	Multiplication of shoots and regeneration of plants	Plantform temporary immersion cultures	Cuenca et al. (2017)
*Bambusa vulgaris*	Multiplication of shoots and regeneration of plants	Twin-flask temporary immersion system	Garcia-Ramirez et al. (2019)
*Musa* sp. Cultivar Rasthali	Multiplication of shoots and rooting of shoots	Balloon-type temporary immersion cultures	Uma et al. (2021)
*Rosa rugosa*	Multiplication of shoots and rooting of shoots	Balloon-type bubble bioreactors (with net/raft)	Jang et al. (2016)
*Alnus glutinosa*	Multiplication of shoots and rooting of shoots	RITA and plantform temporary immersion cultures	San Jose et al. (2020)
*Agave angustifolia* ‘Bacaanora’	Multiplication of shoots and rooting of shoots	RITA temporary immersion cultures	Monja-Mio et al. (2021)
*Salix viminalis*	Multiplication of shoots and rooting of shoots	RITA and plantform temporary immersion cultures	Regueira et al. (2018)
*Salix viminalis*	Multiplication of shoots and rooting of shoots	RITA temporary immersion cultures	Gago et al. (2021)
*Chrysanthemum × morifolium* Ramat. ‘Golden bel’	Multiplication of shoots and rooting of shoots	Temporary immersion cultures (SETIS)	Hwang et al. (2022)
*Fragaria × ananassa* Duch. ‘Seolhyang,’	Multiplication of shoots and rooting of shoots	Temporary immersion cultures (SETIS)	Hwang et al. (2022)
*Cnidium officinale* Makino (*Ligusticum officinale* (Makino) Kitag.	Multiplication of shoots and rooting of shoots	Temporary immersion cultures (SETIS)	Hwang et al. (2022)

### 3.3 Factors affecting the production of somatic embryos

Gaseous environment, shear stress, and light are major factors that affect the formation and culture of somatic embryos in small-scale cultures and bioreactors (Fei and Weathers, 2016). Oxygen, carbon dioxide, and ethylene are the major gases that affect the induction, differentiation, and maturation of somatic embryos (Shigeta et al., 1996; El Meskaoui and Tremblay, 2001; Fisichella and Morini, 2003). A comparatively low oxygen concentration stimulates somatic embryogenesis in wheat (Carman, 1988). In contrast, elevated carbon dioxide (0.3–5%) improves somatic embryo formation (Buddendorf-Joosten and Woltering, 1994; Huang et al., 2006). Ethylene is detrimental during somatic embryogenesis (Jimenez, 2005). Therefore, controlling the gaseous environment of bioreactors during somatic embryo culture is essential. Mixing embryogenic cultures without shear stress is critical for bioreactor cultures. Conventional bioreactors, such as stirred tank bioreactors, create excess shear stress, whereas airlift bioreactors develop foaming within growth chambers; consequently, balloon-type bioreactors are found to be suitable for cultivating somatic embryogenesis (Park et al., 2000; Park et al., 2005; Shohael et al., 2014) in which gaseous environments can be controlled very easily. These bioreactors are attached to cylinders of oxygen, carbon dioxide, and ethylene, and the concentration of these gases is properly controlled and monitored (Jeong et al., 2006; Shohael et al., 2014). Light inhibits the induction and development of somatic embryogenesis. For example, in carrot and Siberian ginseng suspension cultures, darkness induces the production of somatic embryos (Michler and Lineberger, 1987; Shohael et al., 2014). In contrast, red light induces a higher frequency of somatic embryogenesis (D’Onofrio et al., 1998). For coffee, fluorescence is essential, particularly during embryo germination (Ducos et al., 2009). Bioreactor cultures for plant regeneration *via* somatic embryogenesis have been extensively studied (**Table 4**). Temporary immersion RITA, box-in-bag, and temporary root zone immersion bioreactors have been used for regenerating *Coffea arabica, C. canephora*, and *C. arabusta* plants, respectively (Etienne-Barry et al., 1999; Afreen et al., 2002; Ducos et al., 2009). Balloon-type bubble bioreactors have been used for culturing somatic embryos and for subsequent plant regeneration (Park et al., 2000; Park et al., 2005; Shohael et al., 2014). Somatic embryos of orchids, called PLBs, have also been cultured in balloon-type bubble bioreactors for regenerating plants (Park et al., 2000; Yang et al., 2010).

**Table 4:** Selected examples of multiplication of somatic embryos and plant regeneration in bioreactors.

Plant species	Multiplication of somatic embryos and plant regeneration	Type of bioreactors used	References
Somatic embryos			
*Eleutherococcus sessiliflorus*	Multiplication of embryos (from globular to matured stage) and regeneration of plants	Balloon-type bubble bioreactors	Shohael et al. (2005)
*Coffea arabica*	Multiplication of embryos (from globular to matured stage) and regeneration of plants	RITA – temporary immersion bioreactor	Etienne-Barry et al. (1999)
*Coffea arabusta*	Conversion of cotyledonary embryos to plantlets	Temporary root zone immersion bioreactor system (TRI-bioreactor)	Afreen et al. (2002)
*Coffea canephora*	Conversion of cotyledonary embryos to plantlets	Temporary immersion cultures and box-in-bag bioreactors	Ducos et al. (2009)
*Qurcus robour*	Proliferation of embryos	RITA temporary immersion cultures	Mallon et al. (2012)
Protocorm-like bodies (PLBs)			
*Cattleya forbesii*	Multiplication of PLBs	RITA temporary immersion cultures	Ekmekcigil et al. (2019)
*Phalaenopsis* hybrids	Multiplication of PLBs	Balloon-type bubble bioreactors (continuous immersion and temporary immersion)	Park et al. (2000)
*Oncidium* ‘Sweet Sugar’	Multiplication of PLBs	Balloon-type bubble bioreactors (continuous immersion and temporary immersion)	Yang et al. (2010)

## 4 Overcoming the problems of hyperhydricity with plants regenerated in bioreactor cultures

Several decades of studies have demonstrated that liquid cultures are superior to semi-solid cultures for micropropagation of plants because they facilitate fast growth rates, rapid uptake of nutrients by propagules, and involvement of their growth and differentiation. In contrast, the application of bioreactor cultures for micropropagation is useful for large-scale propagation of plants and to reduce production costs (Paek et al., 2005; Etienne and Berthouly, 2002; Georgiev et al., 2014; Mamun et al., 2015). Propagules, such as bulblets, cormlets, pseudobulbs, rhizomes, and microtubers, regenerated in bioreactors do not face the issues of hyperhydricity and can be directly used for *in vivo* transplantation. However, shoots, embryos, and plantlets regenerated in bioreactor cultures (immersion and temporary immersion cultures) are sometimes hyperhydric and show morphological, anatomical, and biochemical variations. This affects up to 30–70% loss of micropropagated plants upon transplantation to *in vivo* conditions (Debnath, 2008; Snyman et al., 2011). Increased ventilation and/or gas supply may control hyperhydricity (Chakrabarty et al., 2003). In addition, the enrichment of carbon dioxide in a bioreactor vessel facilitates the growth of shoots and results in healthy plantlets of sweet potato, potato, and chrysanthemum and Chinese fox gloves (Paek et al., 2001; Paek et al., 2005). Aragon et al. (2010) reported that enhanced light intensity in the presence of carbon dioxide enrichment improved leaf and root growth in *Musa* culture using temporary immersion cultures. Therefore, forced ventilation increases carbon dioxide concentration, and enrichment of light intensity in bioreactors may facilitate the growth of regenerated propagules. Moreover, the usage of CO_2_-enriched air could assist developing propagules in engaging in autotrophy and enhance the physiological and anatomical circumstances of developing plantlets (Xiao et al., 2011; Nguyen et al., 2020; Gago et al., 2021). Additionally, photosynthesis can be improved in bioreactors with forced ventilation, eliminating the need for medium sugars and reducing microbial contamination, producing plants that are physiologically healthier and better suited for acclimatization (Xiao et al., 2011; Nguyen et al., 2020; Rico et al., 2022). **Figure 4** shows examples of the propagation of several plants in bioreactors on a large scale.

## 5 Genetic stability of plants obtained in bioreactors

Micropropagation using a bioreactor system using liquid cultures is a reliable system for large-scale propagation of plants with genetic uniformity, however, sometimes regenerated plants do exhibit genetic variability and this might be due to various factors. The method of plant regeneration, genotype, age of the culture, explant selection, and culture conditions are all crucial elements for the genetic stability of regenerated plants (Rani and Raina, 2000). Direct regeneration shoots, embryos, and propagules such as bulbs, corms, and protocorms invariably maintain genetic stability compared to callus-mediated regeneration (Dale et al., 2008). Evaluation of the genetic purity of regenerated plants is crucial because both genetic (heritable) and epigenetic (non-heritable) variations are documented with *in vitro* regenerated plants. *In vitro* regenerated plants’ genetic homogeneity has been evaluated at the morphological, physiological, biochemical, and genetic levels. Plants produced from tissue culture have been characterized using DNA fingerprinting and several DNA-based markers. For the genetic analysis of tissue-cultured plants, a variety of newly developed special markers have been used, including amplified fragment length polymorphism (AFLP), sequence-tagged sites (STSs), arbitrarily primed polymerase chain reaction (AP-PCR), DNA amplification fingerprinting (DAE), and inter-simple sequence repeat (ISSR) (Rani and Raina, 2000). For instance, Snyman et al. (2011) evaluated sugarcane plants grown in temporary immersion bioreactors in the field and found many variances during the first six months of growth, such as variations in stem diameter and length, but these differences vanished as culture time was extended. They used AFLP analysis to characterize sugarcane plants that had been micropropagated and reported modest levels of polymorphism (0-0.9%). However, bioreactor-cultivated American ginseng and date palm plants have displayed typical phenotypic traits (Fki et al., 2011; Uchendu et al., 2011).

## 6 Conclusions and future perspectives

In bioreactors, a liquid medium is utilized to culture plant explants for regenerating elite clones. Bioreactors provide ideal conditions for mixing cultured tissues with a culture medium, and maintain optimal culture conditions, such as aeration (oxygen, carbon dioxide, and other essential gases), temperature, pH, and illumination regimes, so that cultured tissues grow and develop for regeneration of plants *via* organogenesis or embryogenesis. During plant regeneration, a particular bioreactor does not meet the needs of all short cultures, and immersion-type bioreactors are useful for the regeneration of bulblets, cormlets, rhizomes, and microtubers. Temporary immersion cultures are ideal for regenerating axillary and adventitious shoots and their subsequent rooting. A single bioreactor does not meet all needs of embryogenic cultures because concrete steps, such as induction, differentiation, development, maturation, and germination of somatic embryos, exist. Consequently, different bioreactors are used for different stages to successfully regenerate plants *via* somatic embryogenesis. Future studies should be focused on the adoption of large-scale cultures based on the plant type and mode of regeneration. For the regeneration of bulblets, cormlets, rhizomes, and microtubers adoption of fed-batch cultures are suitable since many nutrients are consumed by growing propagules on a selective/requirement basis. Additionally, changing the composition of the medium—particularly the level of carbohydrates—can aid in getting the best yield and lowering the cost of growing plantlets. The problem of morphological, anatomical, and physiological abnormalities is a bottleneck for plants regenerated in liquid cultures. Although temporary immersion systems eliminate these issues, modifications, including improved aeration, adequate supplementation of essential gases, and balanced light penetration through bioreactors, are necessary. Containers made of glass or other materials that are efficient in light penetration, proper aeration/supplementation designs, and mixing of cultures without endangering aseptic nature are essential. Containers should also be simplistic for the inoculation of explants, transfer of cultures, maintenance, and harvesting of regenerated propagules. Scope for scale-up is necessary, which may efficiently reduce the cost of production

## Author contributions

HM, KJ, KP, and SP designed the study. HM has written the manuscript. KJ has developed figures. KP and SP supervised research work. All authors contributed to the article and approved the submitted version.

## References

[B1] Afreen F. (2006). “Temporary immersion bioreactor,” in Plant tissue culture engineering. Eds. Gupta S. D. Ibaraki Y. (Dordrecht, The Netherlands: Springer), 187–201. doi: 10.1007/978-1-4020-3694-1_11

[B2] Afreen F. Zohayad S. M. A. Kozai T. (2002). Photoautotrophic culture of *Coffea arabusta* somatic embryos: development of a bioreactor for large-scale plantlet conversion from cotyledonary embryos. Ann. Bot. 90, 21–29. doi: 10.1093/aob/mcf151 12125769 PMC4233855

[B3] Aitken-Christie J. Davies H. E. (1988). Development of a semi-automated micropropagation system. Acta Hortic. 230, 81–88. doi: 10.17660/ActaHortic.1988.230.7

[B4] Akita M. Takayama S. (1994). Induction and development of potato tubers in a jar fermenter. Plant Cell Tiss. Organ Cult. 36, 177–182. doi: 10.1007/BF00037717

[B5] Alabarran J. Bertand B. Lartaud M. Etienne H. (2005). Cycle characteristics in a temporary immersion bioreactor affect regeneration, morphology, water, and mineral status of coffee (*Coffea arabica*) somatic embryos. Plant Cell Tiss. Organ Cult. 81, 27–36. doi: 10.1007/s11240-004-2618-8

[B6] Alvard D. Cote T. Teisson C. (1993). Comparison of methods of liquid medium culture for banana micropropagation: effects of temporary immersion of explants. Plant Cell Tiss. Organ Cult. 32, 55–60. doi: 10.1007/BF00040116

[B7] Aragon C. E. Escalona M. Capote I. Pina D. Cejas I. Rodriguez R.. (2005). Photosynthesis and carbon metabolism in plantain (*Musa* AAB) growing in temporary immersion bioreactor (TIB) and *ex-vitro* acclimatization. In Vitro Cell. Dev. Biol. Plant 41, 550–554. doi: 10.1079/IVP2005640

[B8] Aragon C. E. Escalona M. Rodriguez R. Canal M. J. Capote I. Pina D.. (2010). Effect of sucrose, light, and carbon dioxide on plantain micropropagation in temporary immersion bioreactors. In Vitro Cell. Dev. Biol. Plant 46, 89–94. doi: 10.1007/s11627-009-9246-2

[B9] Arigundam U. Variyath A. M. Siow Y. L. Marshall D. Debnath S. C. (2020). Liquid culture for efficient *in vitro* propagation of adventitious shoots in wild *Vaccinium vitis-idaea* ssp. *minus* (lingonberry) using temporary immersion and stationary bioreactors. Sci. Hortic. 264, 109199. doi: 10.1016/j.scienta.2020.109199

[B10] Arnold S. V. Sabla I. Bozhkov P. Dyachok J. Filonova L. (2002). Developmental pathways of somatic embryogenesis. Plant Cell Tiss. Organ Cult. 69, 233–249. doi: 10.1023/A:1015673200621

[B11] Bello-Bello J. J. Canto-Flick A. Balam-Uc E. Gomez-UC E. Robert M. L. (2010). Improvement of *in vitro* proliferation and elongation of habanero pepper shoot (*Capsicum chinense* jacq.) by temporary immersion. HortScience 45, 1093–1098. doi: 10.21273/HORTSCI.45.7.1093

[B12] Buddendorf-Joosten J. M. C. Woltering E. J. (1994). Components of the gaseous environment and their effects on plant growth and development *in vitro* . Plant Growth Regul. 15, 1–16. doi: 10.1007/BF00024671

[B13] Carman J. G. (1988). Improved somatic embryogenesis in wheat by partial simulation of the in-ovulo oxygen, growth-regulator and desiccation environments. Planta 175, 417–424. doi: 10.1007/BF00396349 24221880

[B14] Chakrabarty D. Hahn E. J. Yoon Y. J. Paek K. Y. (2003). Micropropagation of apple root stock M.9 EMLA using bioreactor. J. Hortic. Sci. Biotechnol. 78, 605–609. doi: 10.1080/14620316.2003.11511671

[B15] Correll M. J. Weathers P. J. (2001a). One-step acclimatization of plantlets using a mist reactor. Biotechnol. Bioeng. 73, 253–258. doi: 10.1002/bit.1058 11257608

[B16] Correll M. J. Weathers P. J. (2001b). Effects of light, CO_2_, and humidity on carnation growth, hyperhydration and cuticular wax development in a mist reactor. *In vitro cell. dev* . Biol. Plant 37, 405–413. doi: 10.1007/s11627-001-0071-5

[B17] Cuenca B. Sanchez C. Aldrey A. Bogo B. Blanco B. Correa B.. (2017). Micropropagation of axillary shoots of hybrid chestnut (*Castanea sativa* x *C. crenta*) in liquid medium in a continuous immersion system. Plant Cell Tissue Organ Cult. 131, 307–320. doi: 10.1007/s11240-017-1285-5

[B18] Curtis W. R. (2005). Application of bioreactor design principles to plant micropropagation. Plant Cell Tiss. Organ Cult. 81, 255–264. doi: 10.1007/s11240-004-6646-1

[B19] Curtis W. R. Tuerk A. L. (2008). "Oxygen transport In plant tissue culture systems: Oxygen transport limitations", in Plant tissue culture engineering, Eds. Dutta Gupta S. Ibaraki Y. (Dordrecht: Springer), 173–186. doi: 10.1007/978-1-4020-3694-1_10

[B20] Dale A. Hughes B. R. Donnelly D. (2008). The role of micropropagation in producing specific pathogen-tested plants. HortScience 43, 74–77. doi: 10.21273/HORTSCI.43.1.74

[B21] Debnath S. C. (2008). Developing a scale-up system for the *in vitro* multiplication of thidiazuron-induced strawberry shoots using a bioreactor. Can. J. Plant Sci. 88, 737–746. doi: 10.4141/CJPS07147

[B22] Debnath S. C. (2009). A scale-up system for lowbush blueberry micropropagation using a bioreactor. HortScience 44, 1962–1966. doi: 10.21273/HORTSCI.44.7.1962

[B23] Dewir Y. H. Alsadon A. Al-Aizari A. A. Al-Mohidib M. (2022). *In vitro* floral emergence and improved formation of saffron daughter corms. Horticulturae 8, 973. doi: 10.3390/horticulturae8100973

[B24] D’Onofrio C. Morini S. Bellocchi G. (1998). Effect of light quality on somatic embryogenesis of quince leaves. Plant Cell Tiss. Organ Cult. 53, 91–98. doi: 10.1023/A:1006059615088

[B25] Ducos J. P. Bollon H. Pettard V. (1993). Production of carrot somatic embryos in a bioreactor. Appl. Microbiol. Biotechnol. 39, 465–470. doi: 10.1007/BF00205034

[B26] Ducos J. P. Terrier B. Courtois D. (2009). Disposable bioreactors for plant micropropagation and mass plant cell culture. Adv. Biochem. Eng. Biotechnol. 115, 89–115. doi: 10.1007/10_2008_28 19475375

[B27] Eibl R. Kaiser S. Lombriser R. Eibl D. (2010). Disposable bioreactors: the current state-of-art and recommended applications in biotechnology. Appl. Microbiol. Biotechnol. 86, 41–49. doi: 10.1007/s00253-009-2422-9 20094714

[B28] Eibl R. Werner S. Eibl D. (2009). Disposable bioreactors for plant liquid cultures at litre-scale. Eng. Life Sci. 9, 156–164. doi: 10.1002/elsc.200800102

[B29] Ekmekcigil M. Bayraktar M. Akkus O. Gurel A. (2019). High-frequency protocorm like bodies and shoot regeneration through a combination of thin cell layer and RITA^®^ temporary immersion bioreactor in *Cattleya forbesii* lindl. Plant Cell Tissue Organ Cult. 136, 451–464. doi: 10.1007/s11240-018-1526-2

[B30] El Meskaoui A. Tremblay F. M. (2001). Involvement of ethylene in the maturation of black spruce embryogenic cell lines with different maturation capacities. J. Exp. Bot. 52, 761–769. doi: 10.1093/jexbot/52.357.761 11413212

[B31] Escalona M. Lorenzo J. C. Gonzales B. L. Daquinta M. Borroto k C. G. Gonzales J. I.. (1999). Pineapple (*Ananas cosmos* l. merr) micropropagation in temporary immersion systems. Plant Cell Rep. 18, 743–748. doi: 10.1007/s002990050653

[B32] Espinosa-Leal C. Puente-Garza C. A. Gracia-Lara S. (2018). *In vitro* plant tissue culture: means for production of biological active compounds. Planta 248, 1–18. doi: 10.1007/s00425-018-2910-1 29736623 PMC7088179

[B33] Etienne H. Berthouly M. (2002). Temporary immersion systems in plant micropropagation. Plant Cell Tiss. Organ Cult. 69, 215–231. doi: 10.1023/A:1015668610465

[B34] Etienne-Barry D. Bertrand B. Vasquez N. Etienne H. (1999). Direct sowing of *Coffea arabica* somatic embryos mass-produced in a bioreactor and regeneration of plants. Plant Cell Rep. 19, 111–117. doi: 10.1007/s002990050720 30754735

[B35] Fei L. Weathers P. J. (2014). From cells to embryos to rooted plantlets in a mist bioreactor. Plant Cell Tiss. Organ Cult. 116, 37–46. doi: 10.1007/s11240-013-0380-5

[B36] Fei L. Weathers P. (2016). “Bioreactors for plant embryogenesis and beyond,” in In vitro embryogenesis in higher plants, method in molecular biology. Eds. Germana M. A. Lambardi M. (New York: Springer), 245–258. doi: 10.1007/978-1-4939-3061-6_10 26619865

[B37] Fisichella M. Morini S. (2003). Somatic embryo and root regeneration from quince leaves cultured in ventilated vessels or under different oxygen and carbon dioxide levels. In Vitro Cell. Dev. Biol. Plant 39, 402–408. doi: 10.1079/ivp20033429

[B38] Fki L. Bouaziz N. Kriaa W. Benjamaa-Masmoudi R. Gargouri-Bouzid R. Rival A.. (2011). Multiple bud cultures of ‘Barhee’ date palm (*Phoenix dactylifera*) and physiological status of regenerated plants. J. Plant Physiol. 168, 1694–1700. doi: 10.1016/j.jplph.2011.03.013 21641674

[B39] Gago D. Vilavert S. Bernal M. A. Sanchez C. Aldrey A. Vidal N. (2021). The effect of sucrose supplementation on the micropropagation of *Salix viminalis* l. shoots in semisolid medium and temporary immersion bioreactors. Forests 12, 1408. doi: 10.3390/f12101408

[B40] Gao J. Lee J. M. (1992). Effect of oxygen supply on the suspension cultures of genetically modified tobacco cells. Biotechnol. Prog. 8, 285–290. doi: 10.1021/bp00016a004 1368452

[B41] Gao H. Li J. Ji H. An L. Xia X. (2018). Hyperhydricity -induced ultrastructural and physiological changes in blueberry (*Vaccinium* spp.). Plant Cell Tissue Organ Cult. 133, 65–76. doi: 10.1007/s11240-017-1361-x

[B42] Gao R. Wu S. Q. Piao X. C. Park S. Y. Lian M. L. (2014). Micropropagation of *Cymbidium sinense* using continuous and temporary airlift bioreactor systems. Acta Physiol. Plant 36, 117–124. doi: 10.1007/s11738-013-1392-9

[B43] Georgiev M. I. (2014). “Design of bioreactors for plant cell organ cultures,” in Production of biomass and bioactive compounds using bioreactor technology. Eds. Paek K. Y. Murthy H. N. Zhong J. J. (Dordrecht: Springer), 3–14. doi: 10.1007/978-94-017-9223-3_1

[B44] Georgiev V. Schumann A. Pavlov A. Bley T. (2014). Temporary immersion systems in plant biotechnology. Eng. Life Sci. 14, 607–621. doi: 10.1002/elsc.201300166

[B45] Gogo D. Sanchez C. Aldrey A. Christie C. B. Bernal M. A. Vidal N. (2022). Micropropagation of plum (*Prunus domestica* l.) in bioreactors using photomixotrophic and photoautotrophic conditions. Horticultrurae 8, 286. doi: 10.3390/horticulturae8040286

[B46] Garcia-Ramirez Y. (2023). Temporary immersion system for *in vitro* propagation *via* organogenesis of forest plant species. Trees. doi: 10.1007/s00468-022-02379-w

[B47] Garcia-Ramirez Y. Barrera G. P. Freire-Seijo M. Barbon R. Cancepcion-Hernandez M. Mendoza-Rodriguez M. F.. (2019). Effect of sucrose on physiological and biochemical changes of proliferated shoots of *Bambusa vulgaris* schrad. ex wendl in temporary immersion. Plant Cell Tissue Organ Cult. 137, 239–247. doi: 10.1007/s11240-019-01564-z

[B48] Hahn E. J. Paek K. Y. (2005). Multiplication of chrysanthemum shoots in bioreactors as affected by culture method and inoculation density of single node stems. Plant Cell Tiss. Organ Cult. 81, 301–306. doi: 10.1007/s11240-004-6655-0

[B49] Hao Z. Ouyang F. Geng Y. Geng Y. Deng X. Hu Z.. (1998). Propagation of potato tuber in nutrient mist bioreactor. Biotehcnol. Tech. 12, 641–644. doi: 10.1023/A:1008892332242

[B50] Huang S. Y. Chan H. S. Wang T. T. (2006). Induction of somatic embryos of celery by control of gaseous compositions and other physical conditions. Plant Growth Regul. 49, 219–227. doi: 10.1007/s10725-006-9113-7

[B51] Huang T. K. McDonald K. A. (2009). Bioreactor engineering for recombinant protein production in plant cell suspension cultures. Biochem. Eng. J. 45, 168–184. doi: 10.1016/j.bej.2009.02.008

[B52] Hvoslef-Eide A. K. Olsen O. A. S. Lyngved R. Munster C. Heyerdahl P. H. (2005). Bioreactor design for propagation of somatic embryos. Plant Cell Tiss. Organ Cult. 81, 265–276. doi: 10.1007/s11240-004-6647-0

[B53] Hwang H. D. Kwon S. H. Murthy H. N. Yun S. W. Pyo S. S. Park S. Y. (2022). Temporary immersion bioreactor system as an efficient method for mass production of *in vitro* plants in horticulture and medicinal plants. Agronomy 12, 346. doi: 10.3390/agronomy12020346

[B54] Jang H. R. Lee H. J. Shohael A. M. Park B. J. Paek K. Y. Park S. Y. (2016). Production of biomass and bioactive compounds from shoot cultures of Rosa rugosa using a bioreactor culture system. Hortic. Environ. Biotechnol. 57, 79–87. doi: 10.1007/s13580-016-0111-z

[B55] Jay V. Genestier S. Courduroux J. C. (1992). Bioreactor studies on the effect of dissolved oxygen concentrations on growth and differentiation of carrot (*Daucus carota* l.) cell cultures. Plant Cell Rep. 11, 605–608. doi: 10.1007/BF00236382 24213361

[B56] Jeon S. B. Kan S. W. Kim W. S. Lee G. P. Kim S. H. Seo S. G. (2009). *In vitro* plant regeneration from axillary buds of *Hibiscus syriacus* l. J. Plant Biotechnol. 2, 174–178. doi: 10.5010/JPB.2009.36.2.174

[B57] Jeong C. S. Chakarabarty D. Hahn E. J. (2006). Effects of oxygen, carbon dioxide and ethylene on growth and bioactive compound production in bioreactor culture of ginseng adventitious roots. Biochem. Eng. J. 27, 252–263. doi: 10.1016/j.bej.2005.08.025

[B58] Jimenez V. M. (2005). Involvement of plant hormones and plant growth regulators on *in vitro* somatic embryogenesis. Plant Growth Regul. 47, 91–110. doi: 10.1007/s10725-005-3478-x

[B59] Jimenez E. Perez N. de Feria M. Barbon R. Capote A. Chavez M.. (1999). Improved production of potato microtubers using a temporary immersion system. Plant Cell Tiss. Organ Cult. 59, 19–23. doi: 10.1023/A:1006312029055

[B60] Jin M. Y. Piao X. C. Xiu J. R. Park S. Y. Lian M. L. (2013). Micropropagation using a bioreactor system and subsequent acclimatization of grape rootstock ‘5BB’. Sci. Hortic. 164, 35–40. doi: 10.1016/j.scienta.2013.09.004

[B61] Jo E. A. Murthy H. N. Hahn E. J. Paek K. Y. (2008). Micropropagation of *alocasia amazonica* using semisolid and liquid cultures. In Vitro Cell. Dev. Biol. Plant 44, 26–32. doi: 10.1007/s11627-007-9081-2

[B62] Kamarainen-Karppinen T. Virtanen E. Rokka V. M. Pirttila A. M. (2010). Novel bioreactor technology for mass propagation of potato microtubers. Plant Cell Tiss. Organ Cult. 101, 245–249. doi: 10.1007/s11240-010-9679-7

[B63] Kim N. Y. Hwang H. D. Kim J. H. Kwon B. M. Kim D. Park S. Y. (2020). Efficient production of virus-free apple plantlets using the temporary immersion bioreactor system. Hortic. Environ. Biotechnol. 61, 779–785. doi: 10.1007/s13580-020-00257-3

[B64] Kim E. K. Hahn E. J. Murthy H. N. Paek K. Y. (2004). Enhanced shoot and bulblet proliferation of garlic (*Allium sativum* l.) in bioreactor system. J. Hortic. Sci. Biotechnol. 79, 818–822. doi: 10.1080/14620316.2004.11511848

[B65] Kim Y. J. Wyslouzil B. E. Weathers P. J. (2002). Secondary metabolism of hairy root cultures in bioreactors. In Vitro Cell. Dev. Biol. Plant 38, 1–10. doi: 10.1079/IVP2001243

[B66] Kuzma L. Bruchajzer E. Wysokinska H. (2009). Methyl jasmonate effect on diterpenoid accumulation in *Salvia sclarea* hairy root culture in shake flasks and sprinkle bioreactor. Enzyme Microb. Technol. 44, 406–410. doi: 10.1016/j.enzmictec.2009.01.005

[B67] Leathers R. R. Smith M. A. L. Aitken-Christie J. (1995). “Automation of the bioreactor process for mass propagation and secondary metabolism,” in Automation and environmental control in plant tissue culture. Eds. Aitken-Christie J. Kozai T. Smith M. A. L. (Dordrecht: Springer), 187–214. doi: 10.1007/978-94-015-8461-6_9

[B68] Lee Y. I. Hsu S. T. Yeung E. C. (2013). Orchid protocorm-like bodies are somatic embryos. Am. J. Bot. 100, 2121–2131. doi: 10.3732/ajb.1300193 24136821

[B69] Lema-Ruminska J. Goncerzewicz K. Garbriel M. (2013). Influence of abscisic acid and sucrose on somatic embryogenesis of cactus *Copiapoa tenuissima* ritt. forma *mostruosa* . Sci. World J. 2013, 513985. doi: 10.1155/2013/513985 PMC3694557 23843737

[B70] Lian M. L. Chakarabarty D. Paek K. Y. (2003a). Bulblet formation from bulbscale segments of *Lilium* using bioreactor system. Biol. Plant 46, 199–203. doi: 10.1023/A:1022890208500

[B71] Lian M. L. Chakrabarty D. Paek K. Y. (2002). Growth and uptake of sucrose and mineral ionsby bulblets of *Lilium* oriental hybrid ‘Casablanca’ during bioreactor culture. J. Hortic. Sci. Biotechnol. 77, 253–257. doi: 10.1080/14620316.2002.11511488

[B72] Lian M. L. Murthy H. N. Paek K. Y. (2003b). Photoautotrophic culture conditions and photosynthetic photon flux influence growth of *Lilium* bulblets *in vitro* . In Vitro Cell. Dev. Biol. Plant 39, 532–535. doi: 10.1079/IVP2003440

[B73] Lian M. L. Piao X. C. Park S. Y. (2014). “Mass production of lilium bulblets in bioreactors,” in Production of biomass and bioactive compounds using bioreactor technology. Eds. Paek K. Y. Murthy H. N. (Dordrecht: Springer), 389–415. doi: 10.1007/978-94-017-9223-3_16

[B74] Lim S. Seon J. H. Paek K. Y. Son S. H. Han B. H. (1998). Development of pilot scale process for mass production of *Lilium* bulblets *in vitro* . Acta Hortic. 461, 237–242. doi: 10.17660/ActaHortic.1998.461.25

[B75] Maene P. Debergh P. (1985). Liquid medium additions to established tissue cultures to improve elongation and rooting *in vivo* . Plant Cell Tiss. Organ Cult. 67, 25–35. doi: 10.1007/BF00033566

[B76] Mallon R. Covelo P. Vieitez A. M. (2012). Improving secondary embryogenesis in *Quercus robur*: application of temporary immersion for mass propagation. Trees 26, 731–741. doi: 10.1007/s00468-011-0639-6

[B77] Mamun N. H. Egertsdotter U. Aidun C. K. (2015). Bioreactor technology for clonal propagation of plants and metabolite production. Front. Biol. 10, 177–193. doi: 10.1007/s11515-015-1355-1

[B78] Michler C. Lineberger R. D. (1987). Effects of light on somatic embryo development and abscisic levels in carrot suspension cultures. Plant Cell Tiss. Organ Cult. 11, 189–207. doi: 10.1007/BF00040425

[B79] Monja-Mio K. M. Olvera-Casanova D. Herrera-Alamillo M. A. Sanchez-Teyer F. L. Robert M. L. (2021). Comparison of conventional and temporary immersion systems on micropropagation (multiplication phase) of *Agave angustifolia* haw. ‘Bacanora’. 3 Biotech. 11, 77. doi: 10.1007/s13205-020-02604-8 PMC7810801 33505832

[B80] Mukherjee P. Husain N. Misra S. C. Rao V. S. (2010). *In vitro* propagation of a grape rootstock, deGrasset (*Vitis champinni* planch.): effects of medium compositions and plant growth regulators. Sci. Hortic. 126, 13–19. doi: 10.1016/j.scienta.2010.06.002

[B81] Nguyen Q. T. Xiao Y. Kozai T. (2020). “Photoautotrophic micropropagation,” in Plant factory-an indoor vertical forming system for efficient quality food production, 2nd Edn. Eds. Kozai T. Niu G. Takagaki M. (New York, NY: Elsevier, Academic Press), 333–346. doi: 10.1016/B978-0-12-816691-8.00023-6

[B82] Niemenak N. Saare-Surminski K. Rohsius C. Ndoumou D. O. Lieberei R. (2008). Regeneration of somatic embryos in *Theobroma cacao* L. in temporary immersion bioreactor and analyses of free amino acids in different tissue. Plant Cell Rep. 27, 667–676. doi: 10.1007/s00299-007-0497-2 18193427

[B83] Orozco-Ortiz C. Sanchez L. Araya-Mattey J. Vargas-Solorzano I. Araya-Valverde E. (2023). BIT^®^ bioreactor increases *in vitro* multiplication of quality shoots in sugarcane (*Saccharum* spp. variety LAICA 04-809). Plant Cell Tissue Organ Cult. 152, 115–128. doi: 10.1007/s11240-022-02392-4

[B84] Paek K. Y. Chakrabarty D. Hahn E. J. (2005). Application of bioreactor systems for large scale production of horticultural and medicinal plants. Plant Cell Tiss. Organ Cult. 81, 287–300. doi: 10.1007/s11240-004-6648-z

[B85] Paek K. Y. Hahn E. J. Son S. H. (2001). Application of bioreactors for large-scale micropropagation systems of plants. In Vitro Cell. Dev. Biol. Plant 37, 149–157. doi: 10.1007/s11627-001-0027-9

[B86] Paek K. Y. Murthy H. N. (2002). High frequency of bulblet regeneration from bulb scale sections of *Fritillaria thunbergii* . Plant Cell Tiss. Organ Cult. 68, 247–252. doi: 10.1023/A:1013952803887

[B87] Park S. Y. Ahn J. K. Lee W. Y. Murthy H. N. Paek K. Y. (2005). Mass production of *Eleutherococcus koreanum* plantlets *via* somatic embryogenesis from root cultures and accumulation of eleutherosides in regenerants. Plant Sci. 168, 1221–1225. doi: 10.1016/j.plantsci.2004.12.023

[B88] Park J. A. Park B. J. Kim A. H. Park S. Y. Paek K. Y. (2015). Airlift bioreactor system and nitrogen sources for biomass and antioxidant compound production from *in vitro* culture of Vitis flexuosa plantlets. Hort. Environ. Biotechnol., 56, 358–365. doi: 10.1007/s13580-015-0006-4

[B89] Park S. Y. Murthy H. N. Paek K. Y. (2000). Mass multiplication of protocorm-like bodies using bioreactor system and subsequent plant regeneration in *Phalaenopsis* . Plant Cell Tiss. Organ Cult. 63, 67–72. doi: 10.1023/A:1006420116883

[B90] Pena-Ramirez Y. J. Juarez-Gomez J. Gomez-Lopez L. Jeronimo-Perez J. L. Gracia-Shesena I. Gonzalez-Rodriguez J. A.. (2010). Multiple adventitious shoot formation in Spanish red cedar (*Cedrela odorata* l.) cultured *in vitro* using juvenile and mature tissues: an improved micropropagation protocol for a highly valuable tropical tree species. In Vitro Cell. Dev. Biol. Plant 46, 149–160. doi: 10.1007/s11627-010-9280-0

[B91] Piao X. C. Chakrabarty D. Hahn E. J. Paek K. Y. (2003). A simple method for mass production of potato microtubers using a bioreactor system. Curr. Sci. 84, 1129–1132.

[B92] Rani V. Raina S. N. (2000). Genetic fidelity of organized meristem-derived micropropagated plants: A critical reappraisal. In Vitro Cell. Dev. Biol. Plant 36, 319–330. doi: 10.1007/s11627-000-0059-6

[B93] Regueira M. Rial E. Blanco B. Bogo B. Aldrey A. Correa B.. (2018). Micropropagation of axillary shoots of *Salix viminalis* using a temporary immersion system. Trees 32, 61–71. doi: 10.1007/s00468-017-1611-x

[B94] Rico S. Garrido J. Sanchez C. Ferreiro-Vera C. Codesido V. Vidal N. (2022). A temporary immersion system to improve *Cannabis sativa* micropropagation. Frot. Plant Sci. 13. doi: 10.3389/fpls.2022.895971 PMC9262383 35812929

[B95] Rout G. R. Mohapatra A. Jain S. M. (2006). Tissue culture of ornamental pot plant: A critical review on present scenario and future prospects. Biotechnol. Adv. 24, 531–560. doi: 10.1016/j.biotechadv.2006.05.001 16814509

[B96] Rout G. R. Samantharay S. Das P. (2000). *In vitro* manipulation and propagation of medicinal plants. Biotechnol. Adv. 18, 91–120. doi: 10.1016/s0734-9750(99)00026-9 14538112

[B97] San Jose M. C. Blazquez N. Cernada M. J. Janeiro L. V. Cuenca B. Sanchez C.. (2020). Temporary immersion systems to improve alder micropropagation. Plant Cell Tissue Organ Cult. 143, 265–275. doi: 10.1007/s11240-020-01937-9

[B98] Schuchovski C. Sant’Anna-Santos B. Marra R. Biasi L. (2020). Morphological and anatomical insights into *de novo* shoot organogenesis of *in vitro* ‘Delite rabbiteye’ blueberries. Heliyon 6, e05468. doi: 10.1016/j.heliyon.2020.e54468 33251355 PMC7677692

[B99] Shigeta J. Sato K. J. Mii M. (1996). Effects of initial cell density, pH and dissolved oxygen on bioreactor production of carrot somatic embryos. Plant Sci. 115, 109–114. doi: 10.1016/s0168-9452(96)04327-o

[B100] Shohael A. M. Chakrabarty K. W. Yu K. W. Hahn E. J. Paek K. Y. (2005). Application of bioreactor system for large-scale production of *Eleutherococcus sessiliflorus* somatic embryos in an air-lift bioreactor and production of eleutherosides. J. Biotechnol. 120, 228–236. doi: 10.1016/j.jbiotec.2005.06.010 16095745

[B101] Shohael A. M. Murthy H. N. Paek K. Y. (2014). Pilot-scale culture of somatic embryos of *Eleutherococcus senticosus* in airlift bioreactors for the production of eleutherosides. Biotechnol. Lett. 36, 1727–1733. doi: 10.1007/s10529-014-1534-1 24793494

[B102] Simonton W. Robacker C. Krueger S. (1991). A programmable micropropagation apparatus using cycled medium. Plant Cell Tiss. Organ Cult. 27, 211–218. doi: 10.1007/BF00041292

[B103] Snyman S. J. Nkwanyana P. D. Watt M. P. (2011). Alleviation of hyperhydricity of sugarcane plantlets produced in RITA^®^ vessels and genotypic and phenotypic characterization of acclimated plants. South Afr. J. Bot. 77, 685–692. doi: 10.1016/j.sajb.2011.03.004

[B104] Steingroewer J. Bley T. Georgiev V. Ivanov I. Lenk F. Marchev A.. (2013). Bioprocessing of differentiated plant *in vitro* systems. Eng. Life Sci. 13, 26–38. doi: 10.1002/elsc.201100226

[B105] Szopa A. Kokotkiewicz A. Bednarz M. Jafernik K. Luczkiewicz M. Ekiert H. (2019). Bioreactor types affects the accumulation of phenolic acids and flavonoids in microshoot cultures of *Schisandra chinensis* (Turcz.) baill. Plant Cell Tiss. Organ Cult. 139, 199–206. doi: 10.1007/s11240-019-01676-6

[B106] Takayama S. Akita M. (1994). The types of bioreactors used for shoots and embryos. Plant Cell Tiss. Organ Cult. 39, 147–156. doi: 10.1007/BF00033922

[B107] Teisson C. Alvard D. (1995). “A new concept of plant in vitro cultivation liquid medium: Temporary immersion,” in Current issues in plant molecular and cellular biology. current plant science and biotechnology in agriculture. Eds. Terzi M. Cella R. Falavigna A. (Dordrecht: Spriger), 105–110. doi: 10.1007/978-94-011-0307-7_12

[B108] Teisson C. Alvard D. (1999). *In vitro* production of potato microtubers in liquid medium using temporary immersion. Potato Res. 42, 499–504. doi: 10.1007/BF02358166

[B109] Thul S. T. Kukreja K. (2010). An efficient protocol for high-frequency direct multiple shoot regeneration from internodes of peppermint (*Mentha* x *pierita*). Nat. Prod. Res. 5, 1945–1946. doi: 10.1177/1934578X1000501223 21299127

[B110] Tisserat B. Vandercook C. E. (1985). Development of an automated plant culture system. Plant Cell Tiss. Organ Cult. 5, 107–117. doi: 10.1007/BF00040307

[B111] Trauger M. Hile A. Sreenivas K. Shouse E. V. Bhatt J. Lai T.. (2022). CO_2_ supplementation eliminates sugar-rich media requirement for plant propagation using a simple inexpensive temporary immersion photobioreactor. Plant Cell Tissue Organ Cult. 150, 57–71. doi: 10.1007/s11240-021-02210-3

[B112] Uchendu E. E. Poliyath G. Brwon D. C. W. Saxena P. K. (2011). *In vitro* propagation of north American ginseng (*Panax quinquefolius* l.). In Vitro Cell. Dev. Biol. Plant 47, 710–718. doi: 10.1007/s11627-011-9379-y

[B113] Uma S. Karthic R. Kalpana S. Backyarani S. Saraswathi M. S. (2021). A novel temporary immersion bioreactor system for large scale multiplication of banana (Rasthali AAB-silk). Sci. Rep. 11, 20371. doi: 10.1038/s41598-021-99923-4 34645934 PMC8514489

[B114] Valdiani A. Hansen O. K. Nielsen U. B. Johannsen V. K. Shariat M. Georgiev M. I. Odmidvar V. Ebrahimi M. Tavakoli Dinanai E. Abiri R. (2019). Bioreactor-based advances in plant tissue and cell culture: challenges and prospects. Crit. Rev. Biotechnol. 39, 20–34. doi: 10.1080/07388551.2018.1489778 30431379

[B115] Vidal N. Blanco B. Cuenca B. (2015). A temporary immersion system for micropropagation of axillary shoots of hybrid chestnut. Plant Cell Tissue Organ Cult. 123, 229–243. doi: 10.1007/s11240-015-0827-y

[B116] Vidal N. Sanchez C. (2019). Use of bioreactor systems in the propagation of forest trees. Eng. Life Sci. 19, 896–915. doi: 10.1002/elsc.201900041 32624981 PMC6999064

[B117] Watt M. P. (2012). The status of temporary immersion system (TIS) technology for plant micropropagation. Afr. J. Biotechnol. 11, 14025–14035. doi: 10.5897/AJB12.1693

[B118] Wu S. Q. Lian M. L. Gao R. Park S. Y. Piao X. C. (2011). Bioreactor application on adventitious root culture of *Astragalus membranaceus* . In Vitro Cell. Dev. Biol. Plant 47, 719–724. doi: 10.1007/s11627-011-9376-1

[B119] Xiao Y. Niu G. Kozai T. (2011). Development and application of photoautotrophic micropropagation plant system. Plant Cell Tissue Organ Cult. 105, 149–158. doi: 10.1007/s11240-010-9863-9

[B120] Xu L. Huang H. (2014). Genetic and epigenetic controls of plant regeneration. Curr. Top. Dev. Biol. 108, 1–33. doi: 10.1016/B978-0-12-391498-9.00009-7 24512704

[B121] Yan H. Liang C. Li Y. (2010). Improved growth and quality of *Siraitia grosvenorii* plantlets using a temporary immersion system. Plant Cell Tiss. Organ Cult. 103, 131–135. doi: 10.1007/s11240-010-9752-2

[B122] Yan H. Yang L. Li Y. (2011). Improved growth and quality of *Dioscorea fordii* prain et Burk and *Dioscorea alata* plantlets using a temporary immersion system. Afr. J. Biotechnol. 10, 19444–19448. doi: 10.5897/AJB11.2684

[B123] Yang J. F. Piao X. C. Sun D. Lian M. L. (2010). Production of protocorm-like bodies with bioreactor and regeneration of *in vitro* of *Oncidium* ‘Sugar sweet’. Sci. Hortic. 125, 712–717. doi: 10.1016/j.scienta.2010.05.003

[B124] Yu W. C. Joyce P. J. Cameron D. C. McCown B. H. (2000). Sucrose utilization during potato microtuber growth in bioreactors.. Plant Cell Rep. 19, 407–413. doi: 10.1007/s002990050748 30754795

[B125] Zhang B. Song Bekele L. D. Shi J. Jia Q. Zhang B.. (2018). Optimizing factors affecting development and propagation of *Bletilla striata* in a temporary immersion bioreactor system. Sci. Hortic. 232, 121–126. doi: 10.1016/j.scienta.2018.01.007

